# Automatic modulation classification method using fixed K-means algorithm for feature clustering processing

**DOI:** 10.1371/journal.pone.0333098

**Published:** 2025-10-14

**Authors:** Li Yuan, Yang Chen

**Affiliations:** Department of Mathematics and Computer Science, Hanjiang Normal University, Shiyan, China; The Catholic University of Korea, KOREA, REPUBLIC OF

## Abstract

The role played by communication technology in daily life is gradually increasing. However, there are problems such as complex types of signals, huge amount of data and noise interference, and the recognition accuracy of existing modulation classification methods is low. Therefore, the study proposes a signal automatic modulation classification model based on fixed K-mean algorithm and denoising autoencoder. The model uses fixed K-mean algorithm for feature classification and optimizes median filtering algorithm using dynamic thresholding. The classifier is used to improve the recognition accuracy of specific signals, and long short-term memory and data random corruption denoising are used to optimize the autoencoder. The experiments indicated that the signal classification accuracy of the model were 17.6% and 16.8% higher than the other two models, respectively. The computational complexity of the improved model decreased dramatically, but the average classification accuracy was only 1.6% lower than that before the improvement. The communication overhead and training efficiency were better than the other models, and the number of parameters of the model was 1/2 of that of the pre-improvement model. The memory occupancy and running time were reduced by 335KB and 33ms, respectively. Compared to the other two models, the model’s average classification accuracy at a signal-to-noise ratio of 0 was 18.4% and 19.7% higher, respectively. As a result, the improved model effectively increases signal recognition accuracy, enhances model robustness, and significantly reduces computational complexity while ensuring real-time signal processing for communication computing.

## 1. Introduction

As society continues to develop and progress, communication technology plays an important role in all aspects of life. It greatly improves the efficiency and scope of information transmission, promoting the process of globalization. In civilian fields such as education, entertainment and e-commerce, communication technology also creates huge economic value for society and promotes the prosperity of the market [1,2]. The automatic modulation classification (AMC) technique helps the receiver to determine the signal type for better signal demodulation and decoding. Spectrum management, interference detection, and other domains make extensive use of it [3]. The increasing number of wireless communication services has led to an oversupply of corresponding spectrum resources. Thus, guaranteeing the effectiveness and security of signal demodulation and decoding has emerged as a crucial component in meeting client demands [4]. However, there are problems such as complex types of signals, huge amount of data and noise interference. Therefore, extracting and recognizing the signal type can effectively improve the recognition accuracy (RA) and calculation speed [5]. Existing AMC techniques, such as support vector machines, sparse encoders, convolutional neural network (CNN), etc., suffer from long training time and low RA [6].

With the goal of addressing the issue of AMC performance optimization, Fu X et al. put out a novel compressed automatic machine learning framework to address issues such the high communication cost in the decentralized learning-based AMC approach. The framework adopted the fusion of dispersive learning and integrated learning to realize AMC and reduce the number of model parameters uploading and downloading. Experiments demonstrated that the framework could effectively reduce the communication overhead while maintaining the classification performance of traditional methods [7]. Sun Y et al. proposed a constellation image based classification technique and a feature graphical representation model in order to improve the accuracy of AMC under different signal-to-noise ratios (SNRs). The method was able to detect the modulation type dynamically without phase and frequency locking, represent the statistical features as spider graphs, and test them using software-defined radio simulation and RF data. The method’s categorization accuracy was 59% and 86% at 0dB and 10dB, respectively, according to experiments [8].

To further enhance AMC’s performance, Chang S. et al. suggested a hierarchical model based on a convolutionally gated deep neural network. The model consisted of a three-layer CNN module, two bi-directional gated recurrent units, a hierarchical classification head, and a nonlinear optimal fusion method to generate the fusion weights. Experiments indicated that the model achieved superior performance on public benchmarks using only I/Q displays with low computational overhead [9]. Hao X et al. proposed a new meta-learning method in order to solve the problems such as insufficient labeled signals during AMC. The method constructed a multi-frequency octave to learn low and high frequency features to build meta-tasks related to classification. The study adjusted the normalized distribution to reduce the distributional bias among data and proposed a hybrid method related to classes. Experiments demonstrated that the method was effective in saving computational resources and had superior performance to existing methods [10]. Abdulkarem A M et al. proposed a new two-stage method in order to improve the modulation classification ability under different noise levels. This method combined CNN and continuous wavelet transform and used wavelet transform to extract modulation signal time-frequency information. Experiments demonstrated that the method performed better under different noise levels and was faster in computation [11].

In response to the problem of improving K-means algorithm (KMA), Moodi F et al. proposed a new improved KMA in an attempt the clustering quality of KMA and reduce the computational load. The points whose equidistance threshold exceeded the equidistance index in the distance computation were removed by this procedure. To improve the clustering quality, the distance between the excluded points and the center point of the stable clusters would be included in the calculation again if it was longer than the radius of the clusters. Experiments indicated that this method could improve the clustering quality by 41.85% [12]. Sathyamoorthy M et al. reduced the computational time of clustering algorithm and improved the clustering results by proposing a new KMA. This algorithm deployed nodes into suitable clusters using Q-learning technique for cluster head election. Cluster allocation was carried out in the clustering phase based on the average value, the cluster was divided into k partitions, and cluster head reselection was carried out based on the node energy size. Experiments revealed that the computational delay of this algorithm was reduced by 8.23% and the transmission speed was increased by 1.56% [13]. Gupta M K et al. proposed a new similarity based improved KMA in an attempt to enhance the clustering performance of KMA. A case study was carried out on six datasets, and the algorithm used suitable similarity and distance metrics to improve the performance and accuracy of K-means, mostly employing the Euclidean distance metric [14].

In summary, existing research methods have explored KMA improvement and automatic signal modulation classification from various angles, achieving certain results. However, current methods still have limitations, including lower RA, insufficient robustness, and more complex models. Therefore, an automatic signal modulation classification model based on fixed KMA and denoising selfencoder is proposed. The FK-means algorithm proposed in this study aims to address the issues of local optima and convergence speed caused by the randomness of initial clustering centres. The A-means, K-Means-Q-Learning, and S-K-Mean algorithms in the literature are designed to improve clustering quality, reduce computational latency, and optimise the impact of similarity metrics on clustering performance, respectively. The FK-means algorithm avoids initial centre clustering by randomly selecting the first centre and choosing the farthest point for the second centre, thereby reducing the risk of local optima. The A-means algorithm uses traditional random selection but compensates for the shortcomings of randomness with a dynamic sample removal mechanism. The K-Means-Q-Learning algorithm employs the Q-learning cluster head election strategy, updating the cluster structure based on node energy to reduce computational overhead. The S-K-means algorithm relies on improved distance metrics to enhance performance but does not address the sensitivity of initial centres. The proposed LSTM-DAE model employs a destroy-rebuild mechanism and parameter sharing strategy to reduce computational complexity while maintaining classification accuracy. MCNet uses cascaded multi-scale convolutional kernels to extract features in parallel, thereby improving classification accuracy. MCLDNN adopts a hybrid architecture combining multi-channel CNN branches and LSTM temporal modelling to balance computational efficiency and feature expression capability. MCformer replaces RNN with a Transformer encoder and uses multi-head attention to fuse spatio-temporal features.

The contributions of this study are as follows:(1) This study innovatively determines the initial clustering centers to optimize the KMA and improves the median filtering algorithm by using the dynamic thresholding method. (2) This study fuses long short-term memory (LSTM) and CNN to construct a modulation type recognition module. (3) This study uses a LSTM network and a data stochastic corruption denoising optimized self-encoder to create classifiers to improve the accuracy of specific signal recognition. The research aims to improve the RA of automatic signal modulation classification, reduce the computational complexity of the model, and ensure communication security.

## 2. Methods and materials

### 2.1. Signal feature clustering based on FK-means algorithm

Wireless communication signals need to be denoised before recognition, usually using traditional denoising algorithms or deep learning based denoising algorithms, etc [15]. However, such algorithms may destroy the high SNR signal causing data loss when dealing with noisy signals, leading to a reduction in the final RA [16]. Therefore, the study requires classification processing before denoising the signal, and CNN is used for signal feature (SF) extraction. The CNN has five layers, containing one input layer, two convolutional layers (CLs), one fully connected layer (FCL), and one output layer (OL). The loss function (LF) is calculated as shown in Equation (1).


$$L_{C}=\log\left(1-\log y_{i}\right)\left(1-{\bar{y}}_{i}\right)+\sum_{i=1}^{N}{\bar{y}}_{i}\log y_{i}$$ (1)

In Equation (1), $L_{C}$ denotes the LF. $y_{i}$ is the input data (ID) SNR value. ${\bar{y}}_{i}$ denotes the predicted value of SNR of the ID. N denotes the total number of ID samples. The clustering of SFs is processed using KMA. The ordinary KMA first randomly selects k initial centroids and calculates the clustering of all samples and each initial centroid. The samples are also assigned to the closest centroids to form a cluster respectively. Its centroids are recalculated in the cluster, by iterating until the centroids no longer change. The shortest distance between samples and centroids is calculated as shown in Equation (2) [17].


$$S_{\min}={\left[\sum_{i=1}^{k}\sum_{x_{i}\in R}{\left(x_{j}-P_{i}\right)}^{2}\right]}_{\min}$$ (2)

In Equation (2), $S_{\min}$ denotes the shortest distance between the sample and the center point. $x_{j}$ is the j th sample data. $P_{i}$ denotes the i th center point. Equation (3) displays the standard KMA’s optimization function.


$$f\left(x\right)=\sqrt{\frac{\text{1}}{N}\sum_{i=1}^{N}{\left(x_{i}-P\right)}^{2}}$$ (3)

**Fig 1:**
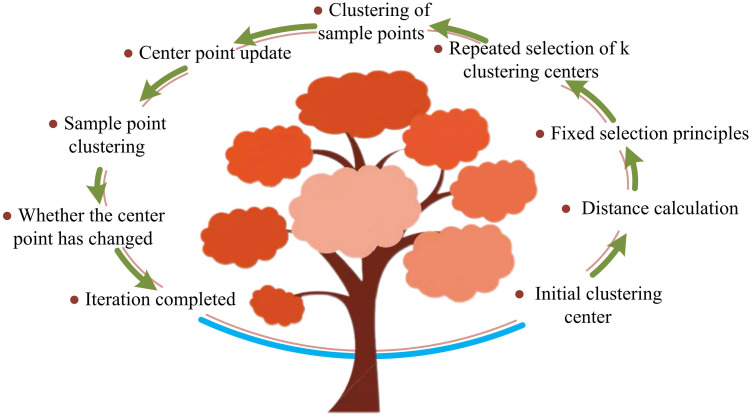
Flow of operation of improved KMA.

In Equation (3), f(x) denotes the optimization function. $x_{i}$ denotes the position of each sample data. P denotes the clustering center point. The running time and clustering effect of the ordinary KMA are affected by the ICC selection, and a wrong selection will significantly reduce the algorithm’s computational speed, and a local optimum situation may also occur [18]. Therefore, this study improves the ICC selection of the KMA by adopting the fixed selection principle. Fig 1 depicts the enhanced KMA’s operational flow.

Fig 1 illustrates the improved KMA. First, it randomly selects a sample from the entire dataset as the initial clustering center. Then, it calculates the distance between the initial center and the remaining samples. Finally, it selects the second center using the fixed selection principle. This process is repeated until k clustering centers are selected. In contrast, the ordinary KMA randomly selects all the clustering centers, making it susceptible to falling into a local optimum. After the selection of clustering centers is completed, the sample cluster classification, centroid update, and final sample clustering are the same as in the ordinary KMA. The fixed selection principle is shown in Equation (4).


$$P_{i}=\frac{\left(S_{\max}-S_{\min}\right)}{\text{2}}$$ (4)

In Equation (4), $S_{\max}$ denotes the maximum distance between the sample and the center point. After the signals are categorized by features, MFA is used to denoise the signals with low SNR. Sorting statistical theory serves as the foundation for the MFA, a nonlinear signal processing computation. The basic idea is to eliminate isolated noise points in a numerical image or digital sequence by replacing the value of a point with the median of the values of nearby points. The effect of the MFA is directly related to the size of the window used. Too small a window will result in insufficient denoising effect, while too large a window will result in loss of information at the edges of the signal. Therefore, the study improves the algorithm by using a dynamic window to enhance the denoising effect. The threshold value of the dynamic window is calculated as shown in Equation (5) [19].


$$T=\left(\frac{\text{1}}{n}\sum_{i=1}^{n}\left|\Delta f\right|+\frac{\text{1}}{n}\sum_{i=1}^{n}\left|\Delta f-\frac{\text{1}}{n}\sum_{i=1}^{n}\left|\Delta f\right|\right|\right)\cdot \frac{f_{ave}}{f_{ave}+f_{var}}$$ (5)

In Equation (5), T denotes the threshold value of the dynamic window. n is the total sample data in the window. Δf is the difference between the center value and the remaining value of the window. $f_{ave}$ is the mean of all data. $f_{var}$ is the standard deviation (SD) of all data. $f_{ave}$ and $f_{var}$ are calculated as shown in Equation (6).


$$\left\{ \begin{array}{c} f_{ave}=\frac{\sum_{i=1}^{n}f\left(x,y\right)}{\text{2}}\\ f_{var}=\sqrt{\frac{\sum_{i=1}^{n}{\left[f\left(x,y\right)-f_{ave}\right]}^{2}}{n}} \end{array}\right.$$ (6)

**Fig 2:**
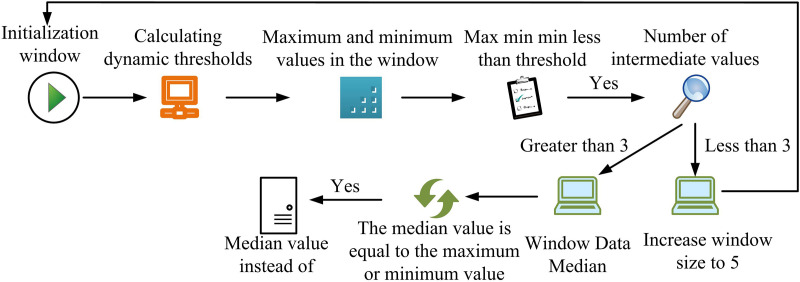
Dynamic window based median filtering algorithm.

In Equation (6), x is the horizontal coordinate of the data within the window. y is the vertical coordinate of the data in the window. The running flow of MFA based on dynamic window is shown in Fig 2.

**Fig 3:**
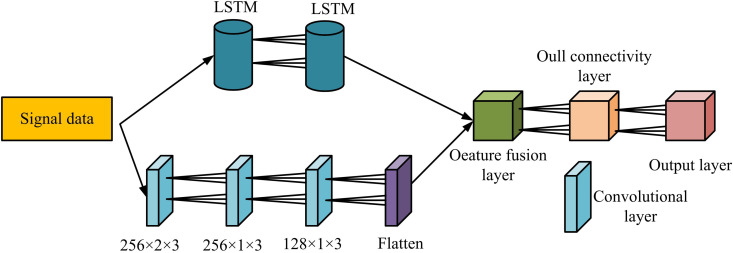
Modulation type recognition module with LSTM and CNN fusion.

Fig 2 shows how to set up an initial window with one row and three columns, as well as how to determine the dynamic threshold and the window’s maximum value (MaxV) and minimum value (MinV). If the MaxV minus MinV is greater than the threshold then the point does not need to be denoised. If not, the data points between the MaxV and MinV is discovered. If the number is greater than 3, calculate the median value within the window. If the number is less than 3, increase the window size to 5. If the median value is equal to the MaxV or MinV then the median value is used instead of it and vice versa no change is made. After the signal denoising is completed, all the high SNR and low SNR signals are subjected to MTR. The MTR module adopts the network structure of LSTM and CNN fusion. The specific architecture is shown in Fig 3.

In Fig 3, the signal samples after processing are completed, and the SFs are extracted in the CL and the LSTM layer, respectively. The two SFs are completely fused in the fusion layer to enhance the completeness of the SFs, ensure the RA of the signal, and reduce the running time. The activation function (AF) of the CL is ReLU, and the multi-dimensional data is compressed into one dimension in the Flatten layer, which is convenient for the subsequent processing in the FCL. The model is feature processed in the FCL and then classified by the OL. The output data is displayed in Equation (7) [20].


$$\left\{ \begin{array}{c} O_{fcl}=f\left(W_{f}O_{c}+b_{f}\right)\\ O_{output}=\sigma \left(W_{o}O_{fcl}+b_{o}\right) \end{array}\right.$$ (7)

In Equation (7), $O_{fcl}$ is the FCL output. f is the Sigmoid AF. $W_{f}$ is the FCL weight value. $O_{c}$ is the feature fusion layer output data. $b_{f}$ is the FCL bias term. $O_{output}$ is the OL data. σ is the Softmax AF. $W_{o}$ is the OL weight value. $b_{o}$ is the OL bias term.

### 2.2. Special signal AMC based on feature networks

FK-means algorithm has been able to effectively classify most of the signals. However, there are some special signals in communication signals. Two types of signals, WBFM, AM-DSB and QAM16, QAM64, have the problem of serious recognition errors. For further improving the RA of signal AMC, this study introduces the signal classification structure and attention mechanism to construct the feature network in the MTR module in the previous section. The study adds WBFM signal classifier and QAM signal classifier to the beginning and end of the MTR module respectively. Attention mechanism is added to the recognition module to extract important features. To emphasize important information and enhance the model’s signal identification and classification abilities, the attention mechanism’s main idea is to let the model selectively focus on various ID components by giving each piece of data a variable weight [21]. The input of the attention structure is the output of the feature fusion layer, and the ID is encoded by the encoder to obtain the output signal, which is a nonlinear function. Then the decoding operation is performed by the decoder along with the weight assignment. The encoder output signal is calculated as shown in Equation (8) [22].


$$a_{n}=f\left(x_{1},x_{2},...,x_{n}\right)$$ (8)

In Equation (8), $a_{n}$ denotes the output signal. f(·) denotes the nonlinear function. $x_{n}$ denotes the ID. The data decoding transportation is shown in Equation (9) [23].


$$\left\{ \begin{array}{c} Y_{1}=f\left(a_{1}\right)\\ Y_{2}=f\left(a_{2},Y_{1}\right)\\ Y_{n}=f\left(a_{n},Y_{1},Y_{2},...,Y_{n-1}\right) \end{array}\right.$$ (9)

In Equation (9), $Y_{n}$ denotes the encoder output signal. The WBFM signal is separated from other signals in the WBFM signal classifier. The differentiation is accomplished by determining the MaxV of this signal’s zero-centered normalized instantaneous amplitude spectral density (NIASD), which is determined using Equation (10).


$$\mu_{\max}=\frac{\max{\left|DFT\left(A_{cn}\right)\right|}^{2}}{N}$$ (10)

In Equation (10), $\mu_{\max}$ denotes the MaxV of the zero-centered NIASD spectral density. N the total number of signal samples and DFT denotes the Fourier transform. $A_{cn}$ denotes the NIASD spectral density of the signal. However, in digital signals, the difference between low and high density signals is also not clear enough. Therefore, the normalized instantaneous frequency kurtosis is used to separate high and low density numerical signals, as displayed in Equation (11) [24].


$$\mu_{42}^{f}=\frac{E\left[f_{N}^{4}\left(i\right)\right]}{E{\left[f_{N}^{2}\left(i\right)\right]}^{2}}$$ (11)

**Fig 4:**
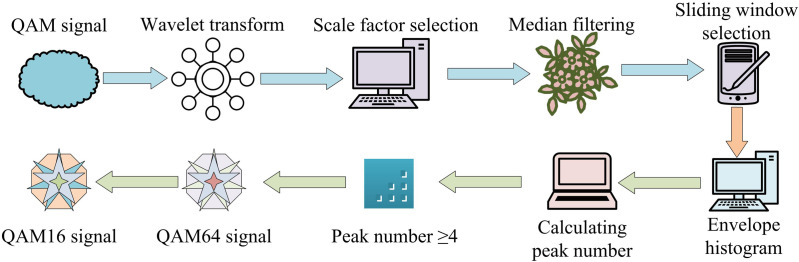
Specific process of QAM classification signal recognition and classification. (Image source: Self-drawn by the author).

In Equation (11), $\mu_{42}^{f}$ denotes the normalized instantaneous frequency kurtosis. E denotes the expected value, i.e., the statistical average over all sampling points. $f_{N}\left(i\right)$ denotes the instantaneous frequency. When the input signal satisfies the zero-centered NIASD spectral density maximum is greater than $\mu_{\max}$ and the normalized instantaneous frequency kurtosis is less than $\mu_{42}^{f}$, the WBFM modulation classification type can be output directly. The signal is then sent to the feature network for additional classification if it is not. The QAM signal classifier needs to determine the characteristics of the signal in the wavelet domain. The specific flow of QAM category signal identification and classification is shown in Fig 4.

**Fig 5:**
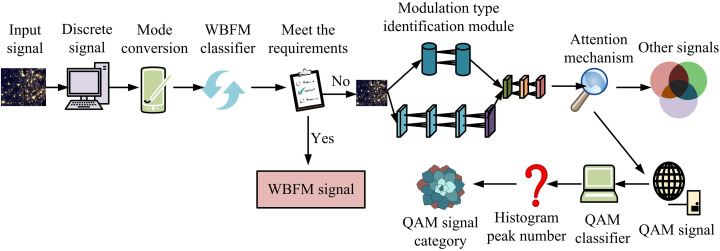
Modulation signal classification algorithm based on feature network. (Image source:https://colorhub.me/photos/Qa2oP).

Fig 4 shows that the input signal class is first detected. If it is a QAM signal, then it undergoes a wavelet transformation. In this case, the wavelet transform scale factor must be less than the sampling frequency divided by the maximum carrier frequency, otherwise there may be a jump point to detect the code elements. After wavelet transforming the signal, median filtering is performed to remove noise interference, and the filtering window needs to be always greater than 5 times the wavelet transform scale factor. After that, the filtered signal envelope histogram is created, and the histogram’s peak count is determined. If the number of peaks is greater than or equal to 4 then the signal type is QAM64 and if the number of peaks is less than 4 then the signal type is QAM16. The structure of modulation signal classification algorithm based on feature network is shown in Fig 5.

In Fig 5, the ID samples are preprocessed first, and then the continuous signals are converted to discrete signals through the sampling process. Then, the discrete signal is converted from I/Q mode to A/P mode to improve the feature network’s ability to extract signal amplitude and phase information. The size of the signal samples is judged in relation to the MaxV of the NIASD and the normalized instantaneous frequency kurtosis of the zero center. Signals that do not satisfy these criteria are input into the modulation type recognition module for further classification processing. Once the other signals have been classified and identified, the QAM signals are input into the classifier to determine the number of histogram peaks for classification.

### 2.3. DAE-based signal AMC

**Fig 6:**
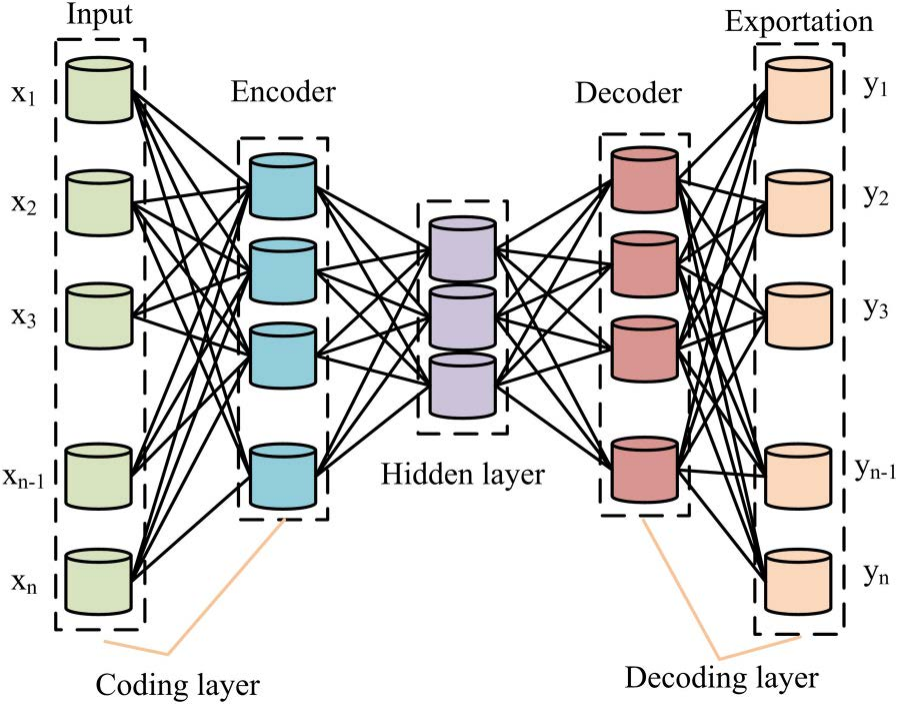
Autoencoder structure.

The MTR module has a longer running time for computation along with a more complex model. To reduce the complexity of signal AMC algorithm and reduce the computation time so that it can be widely used in various low-cost platforms, it is studied that LSTM is used to construct the DAE. This significantly increases the computing speed and lowers the algorithm’s complexity [25]. Before the signal is fed into the DAE, it needs to be subjected to the paradigm regularization process. Controlling the scaling of individual feature values to the same scale eliminates the magnitude problem between features and improves the stability and convergence speed of the algorithm [26]. Paradigm regularization processing by adding a regularization term in the objective function, which is also able to limit the size of the model parameters, thus preventing overfitting. It keeps the model from becoming overly complicated, lessens the overfitting of the training data, and enhances the model’s capacity for generalization [27]. Autoencoder refers to a neural network model that learns an efficient representation of data through unsupervised learning. It does this by setting an error function between the input and output structures, which makes this error function continuously optimized iteratively to finally find the optimal solution. Fig 6 depicts the autoencoder’s construction.

In Fig 6, the autoencoder structure consists of an encoding layer, a hidden layer, and a decoding layer. The hidden layer compresses the ID to a lower dimension by reducing the number of neurons while trying to maintain the features of the original data. This dimensionality reduction process can help remove redundant information from the data and extract the most important features. Meanwhile, the hidden layer can also extract useful feature representations by learning the intrinsic structure of the ID. These features can be used for subsequent machine learning tasks such as classification, clustering, etc. The autoencoder can learn a low-dimensional representation of the data through training. The hidden layer uses the decoder to reconstruct information from the processed data after the data processing is complete. The computation of data in encoder and decoder is shown in Equation (12) [28].


$$\left\{ \begin{array}{c} h_{1}=\sigma_{1}\left(W_{1}x_{1}+b_{1}\right)\\ h_{2}=\sigma_{2}\left(W_{2}x_{2}+b_{2}\right) \end{array}\right.$$ (12)

**Fig 7:**
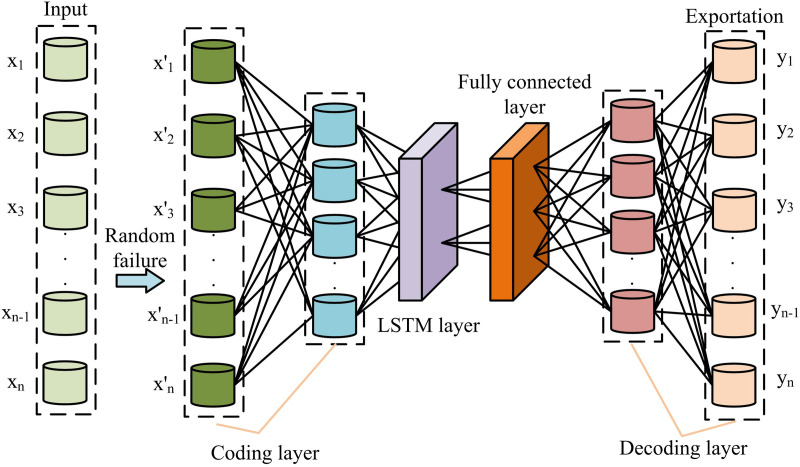
Improved DAE based on LSTM.

In Equation (12), $h_{1}$ is the ID encoding process. $\sigma_{1}$ is the encoder AF. $W_{1}$ is the encoder weights. $x_{1}$ is the ID. $b_{1}$ is the encoder bias term. $h_{2}$ is the output data decoding process. $\sigma_{2}$ is the decoder AF. $W_{2}$ is the decoder weight parameter and $x_{2}$ is the hidden layer output data. $b_{2}$ is the decoder bias term. DAE is improved from autoencoder, which removes noise by randomly destroying the ID, and then performs the related training so as to recover the data. Nevertheless, when recovering the data, DAE might not be able to extract the time series relationship, which results in some data distortion and lowers the signal RA [29]. Therefore, the study uses LSTM, which is superior in processing time series, to improve the DAE. The structure of the improved DAE is shown in Fig 7.

Fig 7 shows an improved denoising self-encoder that replaces the hidden layer of the initial self-encoder with two LSTM layers and two FCLs. The LSTM layer is more suitable for extracting time series features. The two-layer LSTM network acts as an encoder and converts the randomly corrupted ID into state vectors. The two-layer, FCL is used as a decoder. The FCL is used for information downscaling and compression to reduce model complexity. A long short-term memory-denoising autoencoder (LSTM-DAE) based on a LSTM network is constructed. All parameters of the LSTM layer and the FCL can be shared with each other to further reduce the complexity of the algorithm. Equation (13) displays the FCL’s output [30].


$$\left\{ \begin{array}{c} H_{1}=\sigma_{3}\left(W_{3}x_{L}+b_{3}\right)\\ H_{2}=\sigma_{3}\left(W_{4}H_{1}+b_{4}\right) \end{array}\right.$$ (13)

In Equation (13), $H_{1}$ denotes the output of the FCL 1. $\sigma_{3}$ denotes the AF of FCL 1 and FCL 2. $W_{3}$ denotes the weight parameter of the FCL 1. $x_{L}$ denotes the output data of LSTM layer. $b_{3}$ denotes the bias term of the FCL 1. $H_{2}$ denotes the output of FCL 2. $W_{4}$ denotes the weight parameter of full connectivity layer 2. $b_{4}$ denotes the bias term of FCL 2. The LSTM-DAE based LF calculation is shown in Equation (14) [31].


$$L=\left(1-\lambda \right)L_{D}+\lambda L_{F}$$ (14)

In Equation (14), L denotes the LSTM-DAE based LF. λ denotes the balancing parameter. $L_{D}$ denotes data reconstruction loss. $L_{F}$ denotes the classification loss. The balancing parameter of the LF will lead to data loss in autoencoder when it is too large, and the effect of the classification layer may be ignored when it is too small. The predicted SS value is taken as 0.1. The data reconstruction loss and classification loss are calculated as shown in Equation (15).


$$\left\{ \begin{array}{c} L_{D}=\frac{\text{1}}{n}\sum_{j=1}^{n}{\left(x_{j}-{\frac{\mathrm{\backslash buildrel}\mathrm{\backslash lower}3pt\text{\textbackslash{}(\textbackslash{}scriptscriptstyle\textbackslash{}frown\textbackslash{})}}{x}}_{j}\right)}^{2}\\ L_{F}=-\sum_{k=1}^{k}p_{k}\log{\frac{\mathrm{\backslash buildrel}\mathrm{\backslash lower}3pt\text{\textbackslash{}(\textbackslash{}scriptscriptstyle\textbackslash{}frown\textbackslash{})}}{p}}_{k} \end{array}\right.$$ (15)

**Fig 8:**
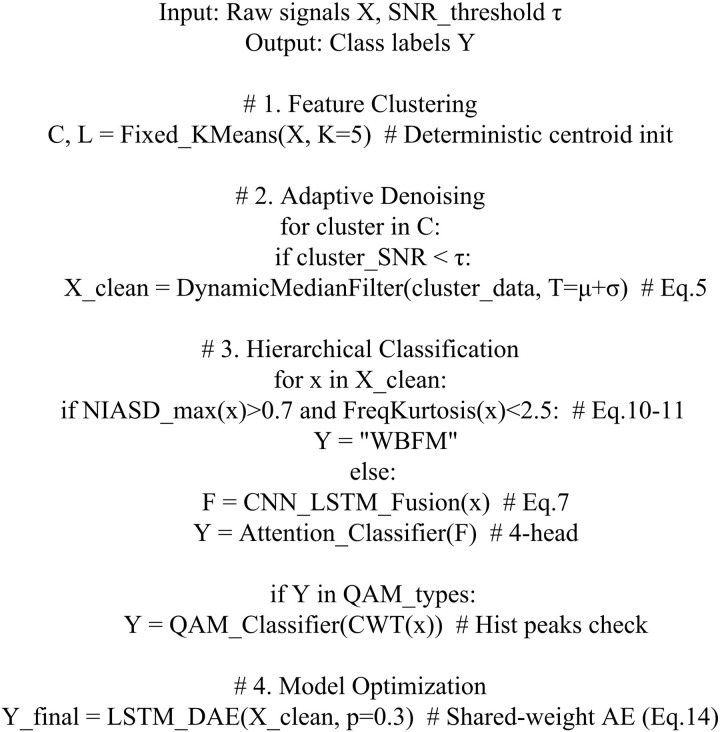
Pseudo-code of signal automatic modulation classification method.

In Equation (15), n denotes the total ID’s quantity. $x_{j}$ the j th ID. S ${\frac{\mathrm{\backslash buildrel}\mathrm{\backslash lower}3pt\text{\textbackslash{}(\textbackslash{}scriptscriptstyle\textbackslash{}frown\textbackslash{})}}{x}}_{j}$ is the j th recovered data. $p_{k}$ denotes the prediction is correct and takes the value of 1. ${\frac{\mathrm{\backslash buildrel}\mathrm{\backslash lower}3pt\text{\textbackslash{}(\textbackslash{}scriptscriptstyle\textbackslash{}frown\textbackslash{})}}{p}}_{k}$ is the likelihood of predicting the ID. The pseudo-code of the signal automatic modulation classification method is shown in Fig 8.

Fig 8 shows the study’s initial processing of the original signal with a fixed KMA and dynamic median filtering for adaptive denoising. The denoised signals are then classified according to a hierarchy. Finally, the improved denoising autoencoder outputs the final signal classification results.

## 3. Results

### 3.1. Experimental analysis of signal AMC based on FK-means algorithm

**Fig 9:**
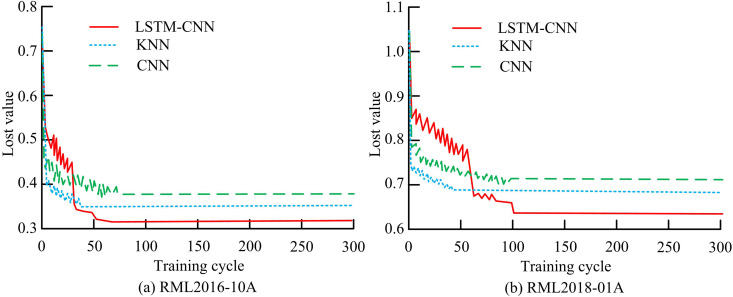
Comparison of loss function curves for different models.

The experimental environment is Inter i7-12600K processor, GPU is GTX 3070Ti, and 32GB of RAM. The datasets used for the study are RML2016-10A and RML2018-01A. The 2016 version contains 8 digital modulation methods and 3 analog modulation methods, with a total sample size of 220k. The 2018 version contains 24 modulation methods with a total sample size of 2,555,904,000. The study divides the two datasets into training and test sets according to the ratio of 8:2, respectively. The learning rate of the feature network during training is 0.001, the discard factor is set to 0.5, the training period is set to 300, the number of attentional heads is 4, and the depth of the attentional layer is 2. The experimental comparison algorithms include LSTM, CNN, and K-nearest neighbors (KNN). Fig 9 illustrates a comparison of the LF curves for several models.

In Fig 9(a), the convergence position of the LSTM-CNN model is 20 times faster than the CNN model and about 15 times slower than the KNN model at 60 iterations. The convergence speed of each model is almost the same in the early stage, rapidly decreasing from 0.75 to 0.5, and there is a curve fluctuation in the middle of the iteration. The loss values of the LSTM-CNN model are 0.06 and 0.03 lower than those of the CNN and KNN models, respectively. In Fig 9(b), as the signal modulation method in the dataset increases, the convergence speed of each model decreases and the convergence position moves further back. The minimum loss value of the LSTM-CNN model has increased, which is 0.07 and 0.04 lower than that of the CNN and KNN models, respectively. The performance comparison of different feature clustering algorithms is shown in Table 1.

**Table 1:** Performance comparison of different feature clustering algorithms.

Algorithm	Contour coefficient	SSE	Number of iterations	Clustering time/ms	Memory usage/M	References
FK-means	0.93	0.52	72	25	12.7	/
A-means	0.90	1.06	93	41	25.6	Reference [12]
K-means-Q-Learning	0.85	1.35	75	29	10.5	Reference [13]
S-K-means	0.91	0.94	118	67	30.2	Reference [14]

**Fig 10:**
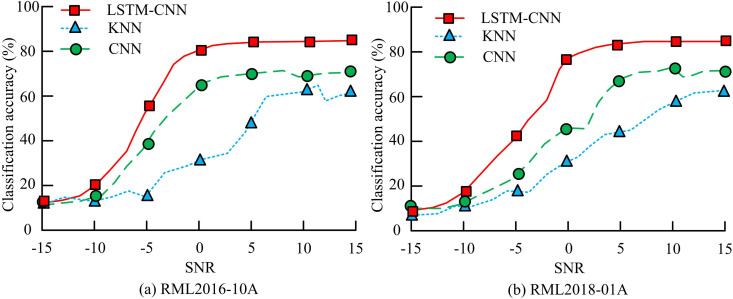
Comparison of signal AMC performance of different algorithms.

In Table 1, the contour coefficient measures the cohesion and separation of the clusters, with higher values being better. The sum of squared errors (SSE) indicates the compactness of the clustering results, with lower values being better. The FK-means algorithm achieves the best clustering performance, outperforming other algorithms across multiple metrics. Its SSE value is 0.52, which is 0.54, 0.83, and 0.42 lower than the improved K-means algorithms proposed in References 12, 13, and 14, respectively. The clustering time for the FK-means algorithm is 25 ms, which is 16 ms, 4 ms, and 42 ms lower than the other three algorithms, respectively. The comparison of signal automatic module classification performance of different algorithms is shown in Fig 10.

In Fig 10(a), the KNN model has poor signal classification accuracy (CA) and large fluctuations in different SNRs, making it unsuitable for processing large-scale data. The LSTM-CNN model has a SNR of −5 and a signal CA of 58.4%, which is 19.2% and 43.5% higher than the KNN and CNN models, respectively. When the SNR is 5, the signal CA of the LSTM-CNN model is 17.6% and 16.8% higher than the other two models, respectively. The model’s CA progressively rises as SNR rises in Fig 10(b). When the SNR is negative, the CA of each model is below 50%. When the SNR is 0, the LSTM-CNN model has a CA of 79.8%, which is 37.5% and 49.6% higher than the KNN and CNN models, respectively. As the modulation type of the dataset signal increases, the CA of each model decreases. However, the LSTM-CNN model has less degradation, indicating its ability to adapt to more complex data. Table 2 displays the modulation CA comparison of several models for various signals when the peak signal-to-noise ratio (PSNR) is 0.

**Table 2:** Comparison of modulation classification accuracy of different signals by different models when the PSNR is 0.

Signal type	LSTM-CNN (%)	CNN (%)	LSTM (%)	KNN (%)
WBFM	51.4	32.5	26.5	12.3
AM-DSB	95.2	91.4	87.5	83.6
QAM-16	85.8	27.0	81.4	37.5
QAM-64	89.6	70.8	82.9	56.7
8PSK	93.5	89.4	91.2	84.0
GFSK	97.5	93.4	96.2	90.4
BPSK	99.8	98.4	99.5	99.0

In Table 2, when PSNR is 0, the CA of the LSTM-CNN model for WBFM signals is 51.4%, which is 18.9%, 24.9%, and 39.1% higher than the CNN, LSTM, and KNN models, respectively. For signals such as AM-DSB, GFSK, BPSK that are easy to classify, the CA of the LSTM-CNN model can reach over 95%. The highest accuracy of BPSK signal is 99.8%. In the QAM-16 signal, the CA of the LSTM-CNN model is 85.8%, which is 58.8%, 4.4%, and 48.3% higher than the other three models, respectively.

### 3.2. Experimental analysis of signal AMC based on improved DAE

**Fig 11:**
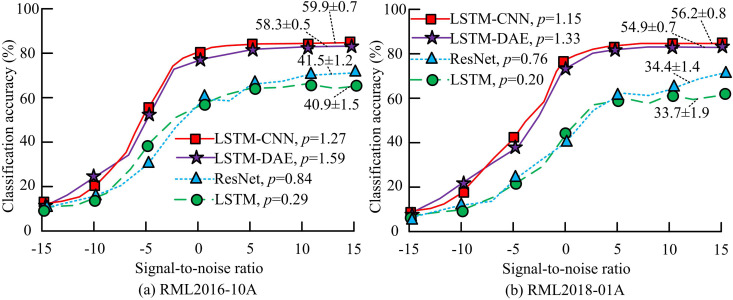
Comparison of signal AMC accuracy of LSTM-DAE model.

The experimental environment is the same as in the previous section, and the quantity of cells in the LSTM is set to 32. The quantity of cells in the two FCLs is 32 and 16. The total number of all signal types output from the OL is seven. The comparison algorithms for the experiments include LSTM-CNN, residual network (ResNet), and LSTM. The accuracy comparison of signal AMC for LSTM-DAE model is shown in Fig 11.

**Fig 12:**
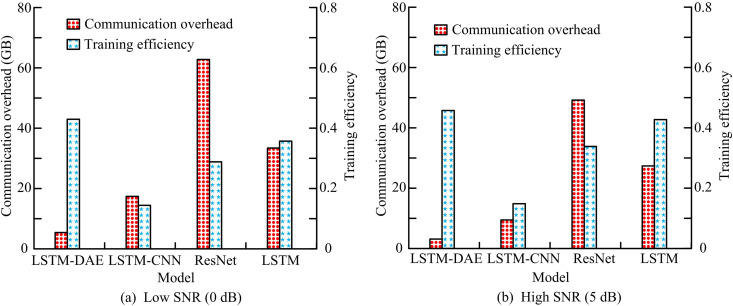
Comparison of communication overhead and training efficiency for different models.

In Fig 11(a), the LSTM-CNN model is improved to reduce its computational complexity. The CA of the improved LSTM-DAE model has decreased. However, among the various SNRs, the average CA has only decreased by 1.6% compared to before the improvement, and the maximum CA can still reach 81.5%. This result is similar to that reported in [30], where a 1–2% decrease in accuracy is considered acceptable in edge deployment when a reduction of >50% in computational overhead is achieved. The experimental results obtained with the LSTM-DAE model are consistent with this industry practice. The average CA of the LSTM-DAE model is 16.8% and 17.4% higher than that of the ResNet and LSTM models, respectively. The standard deviation of the LSTM-DAE model is ± 0.5, which is lower than that of the other three models, indicating that the improved model has high stability. The results of multiple experiments on the LSTM-DAE model are not statistically significant (*p* > 0.05). This indicates that the LSTM-DAE model has high stability, with no significant differences in results across multiple runs. In Fig 11(b), the trend of CA curves for each model remains basically unchanged, but ResNet and LSTM models exhibit significant fluctuations when SNR is high. Its performance decreases when processing large-scale datasets. The average CA of the LSTM-DAE model is 20.5% and 21.2% higher than that of the ResNet and LSTM models, respectively. The comparison of communication overhead and training efficiency among different models is shown in Fig 12.

In Fig 12(a), when the SNR is low, the communication overhead of the LSTM-DAE model is the lowest, which is 12GB, 59GB, and 29GB lower than that of the LSTM-CNN, ResNet, and LSTM models, respectively. The training efficiency of the LSTM-DAE model is defined as 1 divided by the time spent in one training cycle. The training efficiency of the LSTM-DAE model is higher than that of all other models, by 0.28, 0.14, and 0.06, respectively. In Fig 12(b), the communication overhead of the LSTM-DAE model is 1/5 of the original model, which is 48GB and 26GB lower than the ResNet and LSTM models, respectively. The enhanced model’s training efficiency is 0.13 and 0.04 greater than the previous two models, respectively, making it three times more effective than the previous model. Table 3 compares the various models’ levels of complexity.

**Table 3:** Complexity comparison of different models.

Model	Parameter quantity	Number of floating point operations	Memory usage (KB)	Running time (ms)
LSTM-DAE	14685	45037	225	62
LSTM-CNN	28974	95264	560	95
LSTM	200018	663074	2900	185
CNN	5392785	81359725	61500	1260
KNN	257392	576389	2320	230

In Table 3, the LSTM-DAE model has significantly better parameters, floating-point operations, memory usage, and runtime than the other four models. The parameter count of the LSTM-DAE model is half of that of the LSTM-CNN, and its memory usage and running time are 335KB and 33ms lower than those of the LSTM-CNN, respectively. The memory usage is one order of magnitude lower than the remaining three models, and the running time is 123ms, 1192ms, and 168ms lower than LSTM, CNN, and KNN, respectively. Due to the excessive number of CLs, the CNN model has much higher parameter levels than other models, greatly increasing the complexity of the model. Table 4 displays a comparison of the number of signal samples classified per second in various situations.

**Table 4:** Comparison of the number of classification per second of model signal samples in different environments.

Operating environment	Evaluation index	LSTM-DAE	LSTM-CNN	LSTM	CNN
Inter i7-12600K	Mean value	1258	1176	1004	874
	SD	10.8	25.3	19.5	5.4
GTX 3070Ti	Mean value	8529	7284	7395	6508
	SD	152.7	284.6	185.2	100.5
Raspberry Pi 4	Mean value	245	127	45	39
	SD	13.8	5.6	1.2	2.8
Raspberry Pi 3	Mean value	184	52	19	15
	SD	1.5	0.6	1.8	0.4

**Fig 13:**
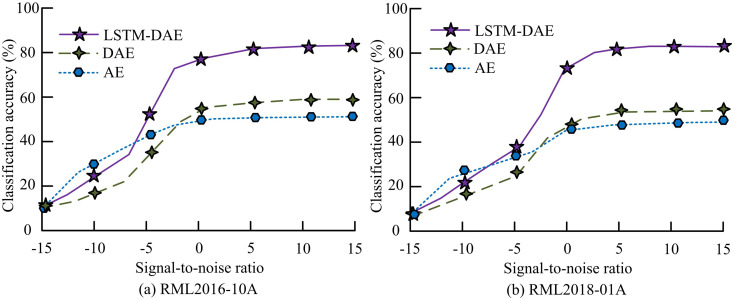
Comparison of LSTM-DAE ablation experiment results.

In Table 4, the experiment compares the signal sample processing capabilities of each model in different operating environments. Using the number of classifications per second as the evaluation metric, a total of 10 experiments are conducted, and the average and SD are compared. When running in the GTX 3070Ti environment, each model classifies the most signals per second. LSTM-DAE is 1245, 1134, and 2021 more than LSTM-CNN, LSTM, and CNN, respectively. The signal classification speed of the model gradually decreases with changes in the operating environment. In the Raspberry Pi 4 system with poor computing power, the LSTM-DAE model has computing speeds 1.93 times, 5.44 times, and 6.28 times faster than the other three models, respectively. In the Raspberry Pi 3 system with the worst computational performance, the LSTM-DAE model has a computation speed 12.27 times faster than the worst CNN model. The LSTM-DAE model has a much lower computational complexity than traditional methods and can effectively run on platforms with poor computing power. The LSTM-DAE ablation experiment is shown in Fig 13.

In Fig 13(a), DAE indicates that the model has removed the LSTM layer, and AE indicates that the model has removed the random fragmentation structure of the ID. At SNR 0, the CA of the LSTM-DAE model is greater than that of the DAE and AE models by 23.5% and 28.4%, respectively, and the average CA is higher than that of the two models by 18.4% and 19.7%, respectively. Fig 13(b) indicates that the accuracy convergence speed of the LSTM-DAE model drops while the changing trends of each model essentially stay the same. However, the MaxV remains unchanged, and the maximum RA of the DAE and AE models has decreased, by 2.7% and 2.5% respectively compared to Fig 13(a). The data random fragmentation and LSTM structure of the LSTM-DAE model can effectively improve the accuracy of signal classification and recognition, and enhance the robustness of the model. The performance comparison of the proposed method with the existing methods is shown in Table 5. The comparison methods include convolutional gated recurrent units deep neural networks (CGDNN), multi-scale attention transfer learning architecture (MATLA), convolutional-LSTM-deep neural network (CLDNN) and visual geometry group (VGG). The study simultaneously introduces several state-of-the-art lightweight AMC models for comparison, including the Multiscale Clustering Network (MCNet), the Multichannel Learning Deep Neural Network (MCLDNN), and the Mixed Channels Transformer (MCformer).

**Table 5:** Comparison of the performance of the proposed method of the study with the existing methods.

Methods	Classification accuracy (%)	Training time (s)	Calculation time (ms)	Time complexity O(n2) evaluation
LSTM-DAE	81.5	2054	62	Lower
CGDNN	80.9	2147	104	Normal
MATLA	81.7	3569	275	Higher
CLDNN	81.2	3094	306	Higher
VGG	75.3	1687	93	Lower
MCNet	76.9	2235	95	Lower
MCLDNN	78.1	2547	124	Normal
MCformer	80.2	2362	159	Higher

**Fig 14:**
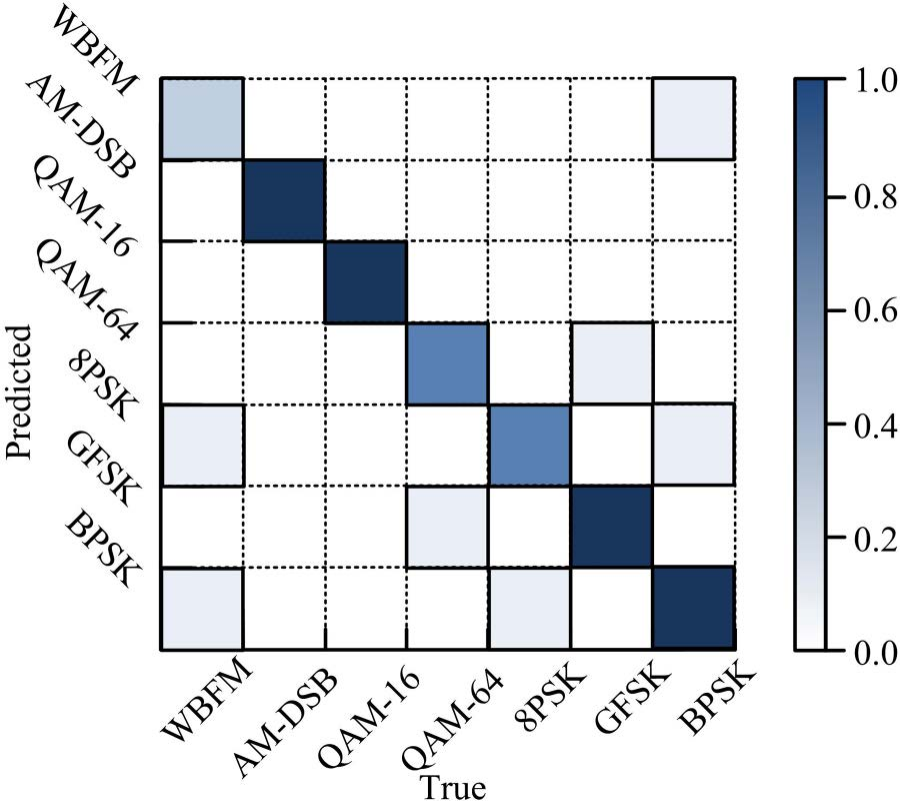
Confusion matrix for different signals at a signal-to-noise ratio of 16.

Table 5 shows that the signal classification accuracy of the LSTM-DAE model is 0.6%, −0.2%, 0.3%, and 6.2% higher than that of the CGDNN, MATLA, CLDNN, and VGG models, respectively. The model requires 2054 seconds to train, which is the lowest computational time among the five methods. Fig 14 shows the confusion matrix of the LSTM-DAE model for different signals at a SNR of 16. Compared with the most advanced lightweight AMC models, the LSTM-DAE model performs better in terms of classification accuracy, training time, and computation time. The classification accuracy of the LSTM-DAE model is 4.6%, 3.4%, and 1.3% higher than that of MCNet, MCLDNN, and MCformer, respectively, while the computation time is 33 ms, 62 ms, and 97 ms lower, respectively.

In Fig 14, when the PSNR is 16 dB, the RA of the four signals (AM-DSB, QAM-16, GFSK, and BPSK) exceeds 99%. The RA of QAM-64 and 8PSK signals can reach 90%, with only a few misdetections and misclassifications. The RA of WBFM signals is 75%, with many misdetections.‌

## 4. Discussion and conclusion

Aiming at the problems of low RA and lack of robustness of existing signal AMC methods, the study proposed a signal AMC model using FK-means algorithm and DAE. The experiments indicated that the convergence position of the LSTM-CNN model had a lower loss value of 0.06 and 0.03 than the CNN and KNN models, respectively, at 60 iterations. The signal CA of the model was 17.6% and 16.8% higher than the other two models, respectively, at an SNR of 5. As the type of signal modulation in the dataset increased, the CA of the models decreased, but the LSTM-CNN model decreased even less. For the signals such as AM-DSB, GFSK, and BPSK, which were easier to classify, the CA of the LSTM-CNN model was able to reach more than 95%. The computational complexity of the LSTM-DAE model decreased dramatically, but the average CA only decreased by 1.6% compared to the pre-improvement period. The communication overhead was 12GB, 59GB, and 29GB lower than the LSTM-CNN, ResNet, and LSTM models, respectively. The training efficiency was 0.28, 0.14, and 0.06 higher than the other three models, respectively. The number of parameters of the model was 1/2 of the pre-improvement number, and the memory occupancy and running time were reduced by 335KB and 33ms, respectively. The computational speed of the model in the worst computationally effective Raspberry Pi 3 system was 12.27 times higher than that of the worst CNN model. It indicated that the computational complexity of the LSTM-DAE model was much lower than that of the traditional method, and was able to run effectively in platforms with poor computational capabilities. The average CA of the LSTM-DAE model was 18.4% and 19.7% higher than the two models when the SNR was 0, respectively. The present study is not without shortcomings. For instance, the MFA exhibits a high removal effect on only a portion of the noise. To enhance the RA of the model, future optimization of the algorithm may entail the incorporation of wavelet threshold denoising.

## Supporting information

S1 File Minimal data set definition. (DOCX)
