# Supplementary material for: Automatic modulation classification method using fixed K-means algorithm for feature clustering processing
Source: PLoS One. 2025 Oct 14;20(10):e0333098. doi: 10.1371/journal.pone.0333098 (PMC12520362; doi:10.1371/journal.pone.0333098)
Supplement: S1 File — (DOCX) [file pone.0333098.s001.docx]

**The data in Figure 9**

| (a) RML2016-10A | | | | (b) RML2018-01A | | | |
| --- | --- | --- | --- | --- | --- | --- | --- |
| Training cycle | Lost value | | | Training cycle | Lost value | | |
|  | LSTM-CNN | KNN | CNN |  | LSTM-CNN | KNN | CNN |
| 50 | 0.33 | 0.35 | 0.38 | 50 | 0.78 | 0.69 | 0.71 |
| 100 | 0.32 | 0.35 | 0.38 | 100 | 0.64 | 0.69 | 0.71 |
| 150 | 0.32 | 0.35 | 0.38 | 150 | 0.63 | 0.68 | 0.71 |
| 200 | 0.32 | 0.35 | 0.38 | 200 | 0.63 | 0.68 | 0.70 |
| 250 | 0.31 | 0.34 | 0.37 | 250 | 0.63 | 0.67 | 0.70 |
| 300 | 0.31 | 0.34 | 0.37 | 300 | 0.63 | 0.67 | 0.70 |

**The data in Figure 10**

| (a) RML2016-10A | | | | (b) RML2018-01A | | | |
| --- | --- | --- | --- | --- | --- | --- | --- |
| SNR | Classification accuracy (%) | | | SNR | Classification accuracy (%) | | |
|  | LSTM-CNN | KNN | CNN |  | LSTM-CNN | KNN | CNN |
| -15 | 11.2 | 11.0 | 10.9 | -15 | 9.5 | 7.2 | 9.7 |
| -10 | 20.1 | 11.4 | 14.3 | -10 | 18.2 | 10.3 | 12.6 |
| -5 | 58.4 | 40.8 | 41.6 | -5 | 40.8 | 23.7 | 18.5 |
| 0 | 80.5 | 31.2 | 64.3 | 0 | 79.8 | 30.2 | 42.3 |
| 5 | 83.7 | 47.6 | 69.8 | 5 | 81.7 | 42.5 | 69.2 |
| 10 | 83.8 | 61.8 | 69.2 | 10 | 82.5 | 59.2 | 74.0 |
| 15 | 84.0 | 61.5 | 71.0 | 15 | 82.5 | 61.3 | 73.5 |

**The data in Figure 11**

| (a) RML2016-10A | | | | | (b) RML2018-01A | | | | |
| --- | --- | --- | --- | --- | --- | --- | --- | --- | --- |
| SNR | Classification accuracy (%) | | | | SNR | Classification accuracy (%) | | | |
|  | LSTM-CNN | LSTM-DAE | ResNet | LSTM |  | LSTM-CNN | LSTM-DAE | ResNet | LSTM |
| -15 | 12.1 | 10.3 | 10.1 | 10.3 | -15 | 9.2 | 8.9 | 8.1 | 8.3 |
| -10 | 20.2 | 22.8 | 17.6 | 16.8 | -10 | 18.5 | 20.7 | 11.5 | 9.4 |
| -5 | 55.2 | 49.9 | 28.5 | 39.1 | -5 | 40.9 | 37.6 | 21.6 | 20.4 |
| 0 | 80.1 | 78.2 | 58.7 | 58.4 | 0 | 78.3 | 72.9 | 39.9 | 42.5 |
| 5 | 83.5 | 81.2 | 65.1 | 64.3 | 5 | 82.5 | 81.1 | 60.4 | 59.2 |
| 10 | 83.5 | 81.4 | 66.2 | 65.2 | 10 | 83.0 | 82.3 | 62.7 | 60.9 |
| 15 | 83.5 | 81.5 | 66.9 | 64.7 | 15 | 83.0 | 82.3 | 71.5 | 61.0 |

**The data in Figure 12**

| (a) Low SNR (0dB) | | | (b) High SNR (5dB) | | |
| --- | --- | --- | --- | --- | --- |
| Model | Communication overhead (GB) | Training efficiency | Model | Communication overhead (GB) | Training efficiency |
| LSTM-DAE | 6 | 0.42 | LSTM-DAE | 2 | 0.45 |
| LSTM-CNN | 18 | 0.14 | LSTM-CNN | 10 | 0.15 |
| ResNet | 65 | 0.28 | ResNet | 50 | 0.32 |
| LSTM | 35 | 0.36 | LSTM | 28 | 0.41 |

**The data in Figure 13**

| (a) RML2016-10A | | | | (b) RML2018-01A | | | |
| --- | --- | --- | --- | --- | --- | --- | --- |
| SNR | Classification accuracy (%) | | | SNR | Classification accuracy (%) | | |
|  | LSTM-DAE | DAE | AE |  | LSTM-DAE | DAE | AE |
| -15 | 10.3 | 10.1 | 10.0 | -15 | 8.9 | 8.3 | 8.5 |
| -10 | 22.8 | 18.5 | 29.4 | -10 | 20.7 | 18.1 | 26.3 |
| -5 | 49.9 | 37.4 | 41.2 | -5 | 37.6 | 26.0 | 33.5 |
| 0 | 78.2 | 54.7 | 49.8 | 0 | 72.9 | 45.0 | 43.2 |
| 5 | 81.2 | 58.4 | 50.1 | 5 | 81.1 | 54.6 | 46.2 |
| 10 | 81.4 | 59.1 | 50.3 | 10 | 82.3 | 55.7 | 47.8 |
| 15 | 81.5 | 59.2 | 50.5 | 15 | 82.3 | 56.5 | 48.0 |

**The data in Figure 14**

| SNR (16dB) | | | |
| --- | --- | --- | --- |
| Signal category | Accuracy rate (%) | False detection rate (%) | Missed detection rate (%) |
| WBFM | 75.4 | 14.3 | 10.3 |
| AM-DSB | 99.5 | 0.3 | 0.2 |
| QAM-16 | 99.1 | 0.8 | 0.1 |
| QAM64 | 92.3 | 6.7 | 1.0 |
| 8PSK | 94.6 | 3.5 | 1.9 |
| GFSK | 100.0 | 0.0 | 0.0 |
| BPSK | 99.4 | 0.2 | 0.4 |
